# Prognostic factors of toxicity of immune checkpoint inhibitors in nonsmall cell lung cancer and small cell lung cancer patients: The ToxImmune study

**DOI:** 10.1002/cnr2.1760

**Published:** 2022-12-09

**Authors:** Francoise Difoum, Antoine Schernberg, Hélène Vanquaethem, Hugo Picchi, Audrey Le Roy, Perrine Vuagnat, Carole Helissey

**Affiliations:** ^1^ Clinical Research unit Military Hospital Begin Saint‐Mandé France; ^2^ Department of Internal Medicine Military Hospital Begin Saint‐Mandé France; ^3^ Department of Medical oncology Military Hospital Begin Saint‐Mandé France

**Keywords:** immune‐related adverse events, immunotherapy, lung cancer

## Abstract

**Background:**

Immunotherapy alone or in combination has clearly improved the survival of patients with lung cancer. However, it may also be responsible for adverse events impacting these patients' quality of life. The ToxImmune study aims to identify prognostic factors that can help to predict immune‐related adverse events.

**Methods:**

We included all patients aged 18 years and older who had received at least one dose of immune checkpoint inhibitors, with or without other therapy, between June 2015 and December 2020 and were diagnosed with nonsmall cell lung cancer or small‐cell lung cancer. Patients' baseline demographic characteristics, biological blood markers, and imaging by PET‐scanner were collected from electronic medical records. All adverse events (AEs) and immune‐related AEs (irAEs) were recorded (Common Terminology Criteria For Adverse Events V.5.0).

**Results:**

Sixty‐four patients were included, of whom 60 (94%) presented at least one irAE. The incidence of Common Terminology Criteria for Adverse Events (CTCAE) grade 2 and grade 3–4 was 34% and 8% respectively.

Female sex, Primitive Tumor Standardized Uptake Value Max (SUVmax) <5, number of metastases ≥3 and immunotherapy received after the first line were found to be significant risk factors for immune‐related adverse events. Based on the number of risk factors, the ToxImmune score predicts the risk of having a grade ≥2 adverse event (primitive tumor SUV ≥ 5 = 0 vs. primitive tumor SUV <5 = 1, number of metastases <3 = 0 vs. number of metastases ≥3 = 1 and L1 = 0 vs. L1 ≥ 1). The incidence of grade ≥2 adverse events was 20%, 55% and 90% with ToxImmune scores 0, 1 and = 2 respectively (*p* = .003). Median progression‐free survival (PFS) times were 19.2 months, 6.64 months and 2.63 months for ToxImmune scores 0, 1 and = 2 respectively, *p* = .13. Median overall survival times were 22.6 months, 16.4 months and 9.8 months for ToxImmune scores 0, 1 and ≥2 respectively, *p* = .24. The disease control rate (DRR) was 78% in ToxIummune score 0 group, and 50% in ToxImmune score 1 and ≥2 groups (*p* = .363).

**Conclusion:**

The ToxImmune score, which is grounded on objective clinical parameters, indicates that cases with a high score had an advanced threat of severe adverse events. The ToxImmune score could therefore be used in clinical practice to identify patients treated for lung cancer with immunotherapy and at risk of severe AE.

## 1 INTRODUCTION

Worldwide, among cancers lung cancer is recognized as the leading cause of cancer death, responsible for 1.8 million deaths (18%) with 2 206 771 new cases diagnosed in 2020.^1^ At the same time, treatment for lung cancers has been revolutionized by the development of Immune Checkpoint inhibitors (ICI), initially in monotherapy and later in combination therapy, which has clearly improved the survival of patients at first in the metastatic stage and today in the localized stage.^2^, ^3^, ^4^, ^5^, ^6^, ^7^, ^8^, ^9^, ^10^, ^11^

By interfering with the immune system, immune check point blockade favors the development of autoimmune complications.^12^, ^13^, ^14^ Activation of T‐cells by ICI can target antigens expressed in normal/nontumoral tissue, or off‐target effects, likely a direct result of breaking immune tolerance, and leading to immunotherapy‐induced adverse effects (AEs).^5^ Immune‐related adverse events (irAEs) are defined as any AE associated with drug exposure and consistent with an immune‐mediated mechanism of action.^15^ Such irAEs are frequent, the incidence can reach 50% in some studies.^6^ Various types of irAEs which have been reported include gastrointestinal, hepatic, endocrine, and skin events, as well as pneumonitis, uveitis, infusion‐related events and fatigue.^16^ The occurrence of irAEs could also be considered as a prognostic factor of response to ICI. Indeed, patients who experienced at least one irAE showed gains in progression‐free survival and overall survival, as well as response rate.^17^, ^18^, ^19^, ^20^, ^21^, ^22^, ^23^ At the same time, these irAEs may have an impact on patients' quality of life and can be life‐threatening. In the event of severe irAEs, which are potentially life‐threatening, the first treatment involves the use of a systemic immunosuppressive treatment, like corticoids, which may counteract the effect of ICIs. Thus, exposure to large amounts of immunosuppressive drugs in the case of high‐grade irAEs would be expected to alter the antitumor effect.^24^

The potential association between irAEs and outcome in patients with nonsmall cell lung cancer (NSCLC), on the one hand, and identification of patients most likely to experience severe irAEs, on the other, are therefore of utmost importance and deserve closer consideration. The ToxImmune study thus aims to provide clinical practitioners with a simple tool, the ToxImmune score, suited for routine practice to help identify patients at risk of severe AE and thereby ensure early medical attention. Moreover, the score could also be taken into consideration as prognostic factor for response.

## 2 PATIENTS AND METHODS

The ToxImmune study was a retrospective study conducted in the Oncology Department at Begin Military Hospital, France, where the study was declared and given prior approval by the local ethics committee.

### 2.1 Patients

We included all patients aged 18 years and older who had received at least one dose of ICI, with or without other treatment, between June 2015 and December 2020 and were diagnosed with NSCLC or small‐cell lung cancer (SCLC). Patients' baseline demographic characteristics, biological blood markers and imaging by PET‐scanner were collected from electronic medical records, as presented below:

### 2.2 Data collection

#### 2.2.1 Patient data

Based on electronic medical records, the following parameters prior to ICI were collected: patient age, sex, smoking history, comorbidities, body weight, height, Body Mass Index (BMI), treatment received before ICI, Eastern Cooperative Oncology Group (ECOG) performance status. Each patient's date of death (if relevant) or date of last follow‐up was recorded as well.

#### 2.2.2 Disease characteristics

The following parameters were collected with regard to disease characteristics: date of diagnosis, histology, programmed death ligand‐1 (PDL1) expression level, mutational status, stage of the disease, number and sites of metastasis, Standardized Uptake Value Max (SUVmax) of primitive tumor.

#### 2.2.3 Biological parameters

Data included complete blood count (CBC), lactate dehydrogenase (LDH), thyroid‐stimulating hormone (TSH), thyroxine (T4), alanine‐ and aspartate‐aminotransferase (ASAT, ALAT), gamma glutamyl transferase (GGT), platelet aggregation level (PAL), Hepatitis B, C, and HIV serology. The platelet‐to‐lymphocyte ratio (PLR) was also calculated accordingly. The Prognostic Nutritional Index (PNI) was calculated as 10 × albumin (g/dl) + 0.005 × lymphocyte count (n/mm^3^), as reported by Onodera et al.^9^ A derived neutrophil to lymphocyte ratio (dNLR) was calculated using the formula *absolute neutrophil count*/(*white blood cell count–absolute neutrophil count*).

#### 2.2.4 Radiological parameters

Baseline SUVmax of primitive tumors before starting ICI treatment was taken into account. We reported the best response on ICI, according iRECIST.

#### 2.2.5 Treatment

Data were collected on: type of ICI received, alone or in combination, dates of start and of end of treatment, number of cycles, reasons for stopping treatment, best response.

#### 2.2.6 Immune adverse events

These include AEs and irAEs (e.g., occurrence, grade, type, treatment and progress). The National Cancer Institute (NCI) of the National Institutes of Health (NIH) has published standardized definitions for adverse events (AEs), known as the Common Terminology Criteria for Adverse Events (CTCAE, also called “common toxicity criteria” [CTC]), to describe the severity of organ toxicity for patients receiving cancer therapy. This topic presents selected tables describing some of the AEs graded in the most recent CTCAE (version 5.0), which was published in November 2017 and became effective in April 2018; it also provides references for other adverse event reporting systems and irAE. Common Terminology Criteria for Adverse Events (CTCAE) v5.0 were used to grade irAEs. For further analyses, AE was divided into grade <2 and ≥2 (severe irAE).

These data were then analyzed with the primary aim of establishing a score, called ToxImmune, which could be used routinely to identify patients at risk of immune AE among those with lung cancer receiving ICI. We defined the ToxImmune score (0; 1; 2) for immune‐oncology (IO) related AE respectively as absence; 1; or ≥2 prognostic factors associated with grade ≥2 AE. The secondary objectives were to assess the impact of these AEs on survival and assess the potential of the ToxImmune score as a prognostic factor for survival and disease control rate (DCR, complete response + partial response and stable disease).

### 2.3 Statistical analysis

Patient and tumor characteristics were compared using the Chi‐squared and Student's *t* tests. The optimal cutoff values as a prognostic variable for ICI toxicities were chosen from a receiver operating characteristic (ROC) curve computing area under the curve (AUC) with the criterion variable and “grade ≥2 AE during ICI treatment” as condition variables.

Survival times were calculated from the time of histological diagnosis and survival rates were estimated using the Kaplan–Meier method. Survival curves were compared using the log‐rank test for the univariate analysis. Time‐to‐event endpoints were defined as the time between the date of first ICI injection and the last follow‐up or first event, recurrence or death for PFS, and estimated by the Kaplan–Meier method. Time‐to‐event curves were compared using the log‐rank test. Hazard ratios (HR) and 95% confidence interval (CI) were estimated with univariate analysis. A *p* value of less than .05 was considered as significant.

Statistical analyses were performed using R (version 3.3.2).

## 3 RESULTS

A total of 64 patients were included in our study and received at least one dose of ICI. The median age was 66 years (range 50–97), 72% were male and 16% presented at least one comorbidity. Fifty patients (72%) had a smoking history. Sixty patients (94%) were diagnosed with nonsmall cell lung cancer and 73% from the entire NSCLC cohort had a metastatic disease. Twenty patients (31%) had PDL1 > 1%. Thirteen patients (20%) presented more than 4 metastatic sites.

The majority of patients (98%) presented an ECOG performance status of 0–1 before starting ICI.

There was a broad range of treatment, primarily immunotherapy alone (75%) (of which, 8% atezolizumab, 75% nivolumab, 17% pembrolizumab), chemotherapy with ICI (22%), or a combination of ICI (3%). The median duration of treatment was 5 months (range 0–60.52). Median follow‐up time was 20.4 months.

Patients' characteristics are summarized in Table 1.

**TABLE 1:** Baseline characteristics of patients with bronchial cancer receiving immunotherapy alone or in combination

	Overall
*n*	64
Median age (range)	66.50 [50, 97]
Sex *n* (%)	
F	18 (28.1%)
M	46 (71.9%)
Median BMI (range)	23.65 [13.77, 46.06]
Tobacco *n* (%)	
Current	18 (28.1%)
Former	32 (50%)
No	14 (21.9%)
Performance Status *n* (%)	
0	22 (34.4%)
1	41 (64.1%)
2	1 (1.6%)
Histology *n* (%)	
Adenocarcinoma	43 (67.2%)
Epidermoid	17 (26.6%)
Small‐cell lung cancer	4 (6.2%)
Mutational status *n* (%)	
EGFR	4 (9.8%)
ALK	2 (5.3%)
KRAS	17 (56.7%)
Median of PDL1 expression (range)	1 [0, 100]
TNM stage *n* (%)	
IB	1 (1.6%)
IIA	2 (3.1%)
IIB	1 (1.6%)
IIIA	6 (9.4%)
IIIB	7 (10.9%)
IVA	29 (45.3%)
IVB	18 (28.1%)
Median SUVmax (range)	9.50 [0, 41.80]
Median number of metastases (range)	2 [0, 7]
Treatment *n* (%)	
CT‐IO	14 (21.9%)
IO	48 (75%)
IO‐IO	2 (3.1%)
Name of Immunotherapy received *n* (%)	
Atezolizumab	17 (26.6%)
Ipilimumab	3 (4.7%)
Nivolumab	33 (51.6%)
Pembrolizumab	11 (17.2%)
Median lines received at inclusion (range)	2 [1, 4]
Biological factors median (range)	
RBC	11.60 [6, 16.70]
Leucocytes	8770 [2710, 24 110]
Neuthophils	5891 [1192, 22 109]
Platelets	276 500 [53 000, 777 000]
Lymphocytes	1534 [290, 3790]
ALAT	21 [5, 98]
ASAT	22 [8, 85]
Creatinine	81 [6.80, 214]
TSH	2 [0.13, 24.09]
T4	14.55 [1, 24.45]
CRP	15.20 [1.10, 320.10]
Albumin	37.20 [22.90, 47.70]
LDH	325 [126, 4780]
NLR	3.90 [0.80, 31.63]
dNLR	2.01 [0.50, 15.40]
PLR	296.82 [51, 1752.14]
PNI	381.05 [232.50, 487.60]

Abbreviations: BMI, body mass index; CT, chemotherapy; dNLR, derived neutrophil‐to‐lymphocyte ratio; F, female; IO, immunotherapy; M, male; NLR, neutrophil lymphocyte ratio; PLR, platelet to lymphocyte ratio; PNI, Prognostic nutritional index; RBC, red blood cell.

Among the 64 patients, 60 (94%) presented least one irAE.

The main irAEs reported were:

- Asthenia in 45% patients after 13.5 median delay from first IO infusion (grade ≥ 2 in 19%),
- Musculoskeletal disorders in 42% after 21 median delay from first IO infusion (grade ≥ 2 in 8%),
- Colitis in 39% after 20 median delay from first IO infusion (grade ≥ 2 in 9%),
- Pneumonitis in 35% after 43 median delay from first IO infusion (grade ≥ 2 in 12%),

There were no cases of grade‐5 AE.

The irAE profile is given in Table 2.

**TABLE 2:** Incidence and profile of irAE

irAE	All grades *n* (%)	≥ grade 2 *n* (%)
Asthenia	29 (45%)	12 (19%)
Musculoskeletal disorders	27 (42%)	5 (8%)
Colitis	25 (39%)	6 (9%)
Pneumonitis	22 (35%)	8 (12%)
Dermatitis	18 (28%)	2 (3%)
Neurological disorders	16 (25%)	4 (6%)
Hematological disorders	11 (17%)	3 (5%)
Thyroid disorders	11 (17%)	2 (3%)
Psychological disorders	9 (15%)	1 (2%)
Nephritis	8 (12%)	1 (2%)
Hepatitis	5 (8%)	3 (5%)
Myocarditis	1 (2%)	0 (0%)

The percentage of patients who stopped immunotherapy due to toxicity was 14% in our population.

The median Progression Free Survival (PFS) was 10.6 months and the median Overall Survival (OS) was 19.3 months. 42% of patients received later‐line treatments after the failure of ICI therapy ICI.

### 3.1 ToxImmune score

In univariate analysis, female sex, primitive tumor SUV max <5, number of metastases ≥3, Line >1, PLR <250 were found to be significantly associated with a risk of AE (*p* < .05) (Table 3).

**TABLE 3:** Comparison of baseline characteristics between patients with grade 0–1 and grade > =2 immune‐related adverse events

	G 0–1	G2‐4	*p*
*N* (%), [range]	37	27	
Age	67 [50, 97]	66 [51, 85]	.913
Sex			
F	6 (16.2%)	12 (44.4%)	.028
BMI	23.97 [16.80, 46.06]	22.65 [13.77, 34.22]	.445
Tobacco			
Current	9 (24.3%)	9 (33.3%)	.449
Former	21 (56.8%)	11 (40.7%)	
No	7 (18.9%)	7 (25.9%)	
Performance status			.515
0	14 (37.8%)	8 (29.6%)	
1	22 (59.5%)	19 (70.4%)	
2	1 (2.7%)	19 (70.4%)	
Histology			
Adenocarcinoma	25 (67.6%)	18 (66.7%)	.350
Epidermoid	11 (29.7%)	6 (22.2%)	
Small‐cell lung cancer	1 (2.7%)	3 (11.1%)	
Mutational status			
EGFR	3 (13.6%)	1 (5.3%)	.709
ALK	1 (4.3%)	1 (6.7%)	1.000
KRAS	10 (55.6%)	7 (58.3%)	1.000
TNM stage			
IB	0 (0%)	1 (3.7%)	.378
IIA	2 (5.4%)	0 (0%)	
IIB	0 (0%)	1 (3.7%)	
IIIA	4 (10.8%)	2 (7.4%)	
IIIB	4 (10.8%)	3 (11.1%)	
IVA	19 (51.4%)	10 (37%)	
IVB	8 (21.6%)	10 (37%)	
SUVmax	12.60 [2.40, 35.60]	5.70 [0, 41.80]	.007
Median number of metastases	2 [0, 7]	3 [0, 4]	.004
Treatment			
CT‐IO	11 (29.7%)	3 (11.1%)	.077
IO	24 (64.9%)	24 (88.9%)	
IO‐IO	2 (5.4%)	0 (0%)	
Name of Immunotherapy received *n* (%)			
Atezolizumab	11 (29.7%)	6 (22.2%)	.144
Ipilimumab	3 (8.1%)	0 (0%)	
Nivolumab	15 (40.5%)	18 (66.7%)	
Pembrolizumab	8 (21.6%)	3 (11.1%)	
Median lines received at inclusion	1 [1, 4]	2 [1, 4]	.010
RBC	10.85 [6, 16.70]	11.70 [8.10, 15.30]	*.229*
Leucocytes	8755 [2710, 24 110]	9000 [2870, 14 870]	*.961*
Neuthophils	5737 [1192, 22 109]	6064 [1660, 10 860]	*.956*
Platelets	271 000 [86 000, 777 000]	279 000 [53 000, 561 000]	*.859*
Lymphocytes	1517 [435, 3350]	1551 [290, 3790]	*.994*
ALAT	23.50 [5, 98]	18 [8, 61]	*.177*
ASAT	23 [8, 85]	22 [11, 42]	*.723*
Creatinine	81 [14.10, 214]	85 [6.80, 162.40]	*.754*
TSH	2 [0.22, 7.37]	1.94 [0.13, 24.09]	*.674*
T4	14.70 [1, 20.04]	14.47 [12.35, 24.45]	*.450*
CRP	18 [1.30, 320.10]	15.20 [1.10, 295]	*.928*
Albumin <38 g/L	12 (54.5%)	9 (81.8%)	*.250*
LDH ≥200	14 (56%)	10 (76.9%)	*.770*
NLR ≥2.5	27 (77.1%)	16 (69.6%)	*.735*
dNLR	1.91 [0.50, 15.40]	2.18 [0.92, 9.11]	*.890*
PLR < 250	7 (21.2%)	13 (54.2%)	*.022*
PNI	385.22 [232.50, 487.60]	378.95 [332.46, 460.44]	*.819*

*Note*: Chi‐squared and Student's *t*‐tests were performed to assess risk factors for AE and irAEs.

Abbreviations: BMI, body mass index; CT, chemotherapy; dNLR, derived neutrophil‐to‐lymphocyte ratio; F, female; IO, immunotherapy; M, male; NLR, neutrophil lymphocyte ratio; PLR, platelet to lymphocyte ratio; PNI, Prognostic nutritional index; RBC, red blood cell.

Based on this analysis, we established the ToxImmune score to predict the risk of having a grade ≥ 2 adverse event (primitive tumor SUV ≥5 = 0 vs. primitive tumor SUV <5 = 1, number of metastases <3 = 0 vs. number of metastases ≥3 = 1 and L1 = 0 vs. > L1 = 1).

Female sex, although significantly associated with higher grades of AE, was not included in the calculation because, statistically, the Chi2 test of independence did not indicate that it could be dissociated from the number of metastases (*p* = .03). Given that, of the two, median number of metastases was more strongly associated with the risk of grade ≥2 AE than sex and more reliably so (*p* = .004 vs. *p* = .028 respectively), the number of metastases, rather than sex, was taken into account in the ToxImmune score, although female sex can still be considered as a complementary indicator.

PLR <250 was also significantly associated with the risk of AE grade ≥2, but given the infrequent use of PLR in clinical routine, it was not included in the ToxImmune score.

This score was defined as:

- (0) absence of any prognostic factor associated with grade ≥2 AE;
- (1) prognostic factor associated with grade ≥2 AE;
- (2) ≥ 2 prognostic factors associated with grade ≥2 AE.

**FIGURE 1:**
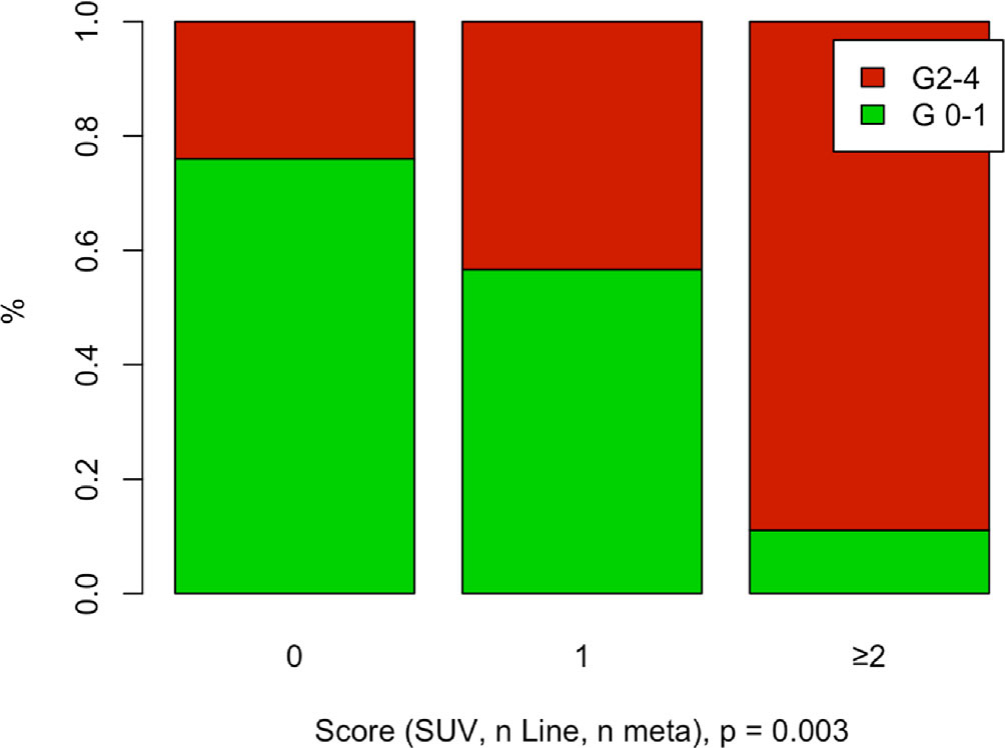
Toxicity by ToxImmune score

The incidence of grade ≥2 adverse events was 24%, 57% and 89% with ToxImmune score 0, 1 and ≥2 respectively, *p* = .003 (Figure 1).

**FIGURE 2:**
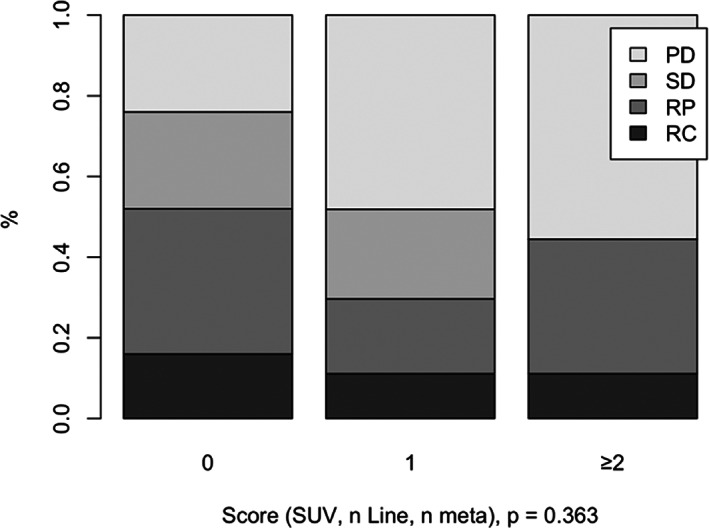
Overall response rate by ToxImmune score. PD, progression disease; RC, complete response; RP, partial response; SD, stable disease

The DCR was 78% in the ToxIummune score = 0 group, 50% in ToxImmune score 1 and 2 groups, (*p* = .363) (Figure 2). Likewise, there was a discernible connection between the ToxImmune score and the risk of “progressive disease” at the first assessment of the disease after initiation of ICI (at week 6): 24% for score = 0, 48% for score = 1, and 56% for score = 2, (*p* = .114).

**FIGURE 3:**
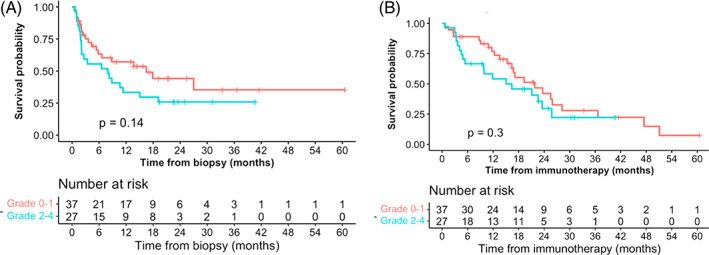
(A) PFS by AE grade. (B) OS by AE grade

Grade ≥2 AEs were linked to a decline in survival rates, both PFS and OS. In patients who experienced grade 0 or 1 AEs, median PFS was 16.5 months, as opposed to 7.9 months in patients who experienced grade ≥2 AEs (*p* = .14) (Figure 3A). Similarly, the median OS was 21.8 months in patients who experienced grade 0 or 1 AEs, but 15.7 months in patients who experienced grade ≥2 AE (*p* = .3) (Figure 3B).

**FIGURE 4:**
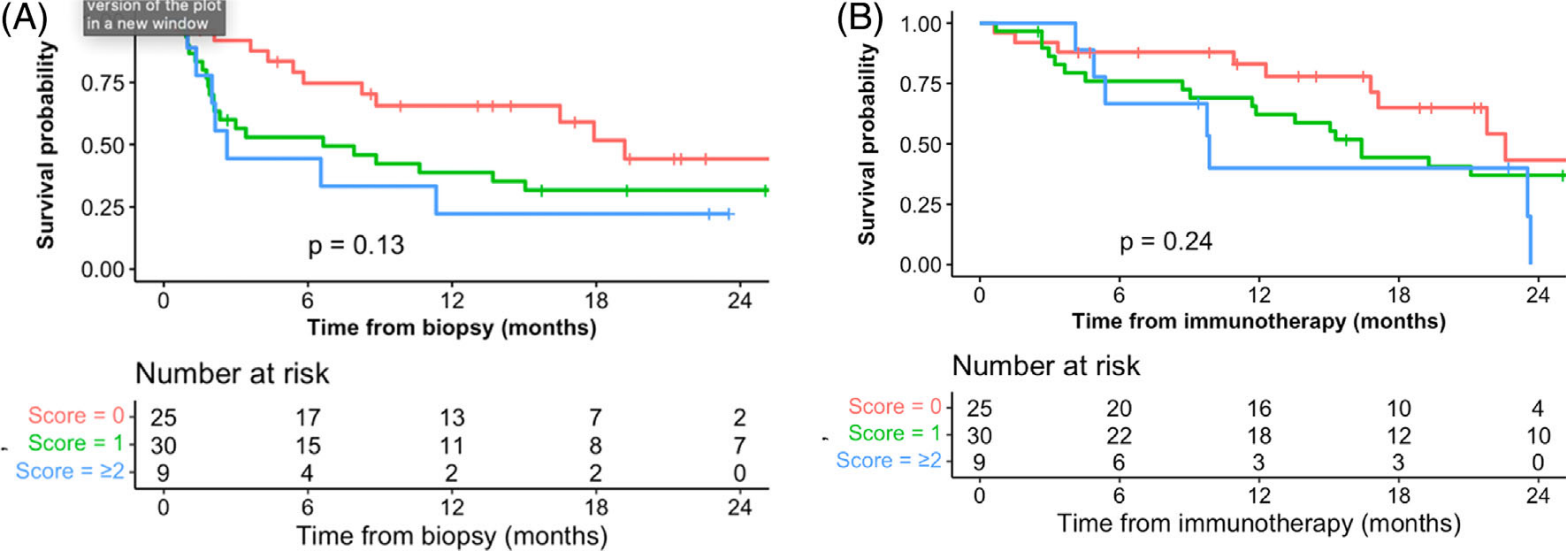
(A) PFS by ToxImmune score. (B) OS by ToxImmune score.

For ToxImmune score 0, 1 and 2, the median PFS was 19.2 months, 6.64 months and 2.63 months respectively, *p* = .13 (Figure 4A), while the median OS was 22.6 months, 16.4 months and 9.8 months, *p* = .24 (Figure 4B).

In addition to predicting the incidence of grade ≥2 AEs, the ToxImmune score thus appears as a more accurate prognostic factor for survival, both PFS and OS, than AEs alone.

## 4 DISCUSSION

To the best of our knowledge, this is the first study establishing a predictive AE score, which can be used in clinical practice to identify patients at high risk of AE and monitor them more closely.

ICI treatment may cause irAEs in any part of the body, thus presenting a serious challenge for clinicians. The reported incidence of any‐grade irAEs associated with ICI treatment ranges from 15% to 90%, depending on the therapeutic class of the ICIs and the type of cancer.^25^ In our study, the incidence of irAEs was 90%, including 58% grade 1. The incidence of severe adverse event was 8%, similar to incidence rates reported elsewhere in the literature. The main AEs and irAEs reported were musculoskeletal disorders followed by digestive disorders, pneumonitis and dermatological disorders.

In accordance with our own findings, Valpione et al confirmed the impact of female sex on the occurrence of adverse events.^26^ They reported that female sex was significantly and independently associated with higher risk of severe AE (OR = 1.5, 95% CI 1.06–2.16). In contrast, however, while both studies point to female sex as a prognostic factor for AE, our data do not show it to be independent from the number of metastases.

Other studies have shown a correlation between irAE and survival. Patients who experienced at least one irAE survived longer compared to patients with no irAE.^8^, ^9^, ^10^, ^11^, ^12^, ^13^, ^14^, ^27^, ^28^, ^29^, ^30^, ^31^, ^32^, ^33^ Our analysis suggests that the severity of irAE has an even greater impact on overall survival. Indeed, patients with irAE grade <2 had numerically greater PFS and OS than those with irAE grade ≥2. Ksienski et al reported that patients who presented irAEs leading to discontinuation of treatment had shorter survival times compared to those who continued treatment (median OS 8.3 vs. 14.5 months, p = .008).^34^ Likewise in the same study, patients who presented irAE grade 3 or higher had shorter survival times with HR = 2.29 (1.05–4.98), *p* = .036.^16^ Socinski et al recently presented at ASCO 2021 an exploratory pooled analysis of IMpower130, 132 and 135.^8^, ^35^, ^36^, ^37^ They reported that patients who experienced at least one irAE had a longer OS, with HR = 0.69 (95% CI: 0.60–0.78). But patients with at least one grade 3–5 irAE had a shorter OS. They attributed these findings to the interruption of the treatment.^8^ It is therefore essential to identify patients at risk of severe AE, in order to maintain the clinical benefit of ICI.

Among other predictive factors, thyroid uptake in FDG PET has been shown to be a predictor of thyroid AE and receiving ICI beyond the first line increased the risk of AE, which may be related to the immunogenic potential of chemotherapy.

Our study is the first to assess the impact of tumor volume and its metabolic character on the incidence of irAEs.

According to our findings, primitive tumor SUV max <5, number of metastases ≥3, Line >1, PLR <250 and female sex (though not independent of the number of metastases) are associated with a risk of AE, while Primitive tumor SUV max <5, number of metastases ≥3 are associated with a significantly higher risk of severe AE. Based on these criteria, the ToxImmune score can help clinicians identify at‐risk patients and set up close monitoring to limit the effects of AE our patients' quality of life.

Grade 1, obviously, is easier to manage than grade 3–4. The primary aim of the ToxImmune score is therefore to predict grade ≥2 adverse events, the incidence of which was 24%, 57% and 89% with ToxImmune scores 0, 1 and 2 respectively. This suggests that patients with a high ToxImmune score should be closely monitored to prevent serious adverse events.

Considering that DCR was 78% in ToxIummune score = 0 group, 50% in ToxImmune score 1 and ≥2 groups, while the median PFS was 19.2 in ToxImmune = 0, 6.64 in ToxImmune = 1 and 2.63 in ToxImmune ≥2 patients, the ToxImmune score, though not statistically significant given the relatively small sample size, could nevertheless be used as a predictor of response to immunotherapy. ToxImmune scores 0, 1 and 2 also appear to be a strong prognostic factor for PFS and OS.

Although our study did not reveal any biological factors significantly associated with the occurrence of adverse events, Diehl et al. reported that that patients with higher baseline lymphocyte counts (>2000) have a greater risk for irAE (*p* < .01).^26^ This could therefore be taken into account, like patients' sex, as a complementary indicator alongside the ToxImmune score for the management of patients initiating ICI. Other biological factors have been reported to be associated with a risk of AE, such as IL6, IL17 but also 11 circulating cytokine biomarkers (G‐CSF, GM‐CSF, fractalkine, fibroblast growth factor‐2, IFN‐α2, IL12p70, IL‐1α, IL‐1β, IL1Rα, IL‐2, and IL‐13) which were significantly upregulated in patients with severe irAEs at baseline and early during treatment.^23^, ^38^, ^39^, ^40^, ^41^ These biomarkers, however, have only a limited role in current practice and were therefore not included in the ToxImmune score to ensure that it could be calculated simply using commonly available information.

We acknowledge the limitations of our study, mainly because of its retrospective nature the sample size with an heterogenous population (different agents used, a mixture of locally‐advanced and metastatic patients, and SCLC and NSCLC patients).

Nevertheless, our data came from a real‐world scenario and provide a basis on which to calculate the ToxImmune score in patients receiving ICI treatment for lung cancer. A validation cohort will be used to assess the suitability and importance of the ToxImmune score as a prognostic factor for immune‐mediated AE and prognostic factor for response to ICI and survival.

## 5 CONCLUSION

ICI has clearly changed the prognosis for patients with metastatic lung cancer and has also shown an impact on survival in localized stages without metastasis. However, the risk of AE impacts patients' quality of life and may prove life‐threatening. It is therefore essential to identify patients at risk of serious AEin order to prevent, identify and manage adverse events early on. The ToxImmune score provides a helpful tool for routine clinical practice and may prove useful in discussing the management of at‐risk patients.

## AUTHOR CONTRIBUTIONS

**Françoise Difoum:** Data curation (equal); formal analysis (equal); resources (equal); writing – original draft (equal); writing – review and editing (equal). **Antoine Schernberg:** Formal analysis (equal); investigation (equal); methodology (equal); resources (equal); supervision (equal); validation (equal); visualization (equal); writing – original draft (equal); writing – review and editing (equal). **Hélène Vanquaethem:** Investigation (equal); writing – original draft (equal); writing – review and editing (equal). **Hugo Picchi:** Investigation (equal); writing – original draft (equal); writing – review and editing (equal). **Audrey Le Roy:** Investigation (equal); writing – original draft (equal); writing – review and editing (equal). **Perrine Vuagnat:** Investigation (equal); writing – original draft (equal); writing – review and editing (equal). **Carole Helissey:** Conceptualization (lead); formal analysis (equal); methodology (lead); project administration (lead); resources (lead); software (lead); supervision (lead); validation (lead); visualization (lead); writing – original draft (lead); writing – review and editing (lead).

## FUNDING INFORMATION

This research received no external funding.

## CONFLICT OF INTEREST

The authors declare no conflict of interest with this work.

## Data Availability

Data sharing is not applicable to this article as no new data were created or analyzed in this study.
